# Adverse events caused by potential drug-drug interactions in an intensive care unit of a teaching hospital

**DOI:** 10.5935/0103-507X.20150060

**Published:** 2015

**Authors:** Mariana Macedo Alvim, Lidiane Ayres da Silva, Isabel Cristina Gonçalves Leite, Marcelo Silva Silvério

**Affiliations:** 1 Universidade Federal de Juiz de Fora - Juiz de Fora (MG), Brazil.

**Keywords:** Drug interactions, Anti-infective agents, Drug-related side effects and adverse reactions, Pharmaceutical preparations, Drug utilization, Hospital, teaching, Intensive care

## Abstract

**Objective:**

To evaluate the incidence of potential drug-drug interactions in an intensive care unit of a hospital, focusing on antimicrobial drugs.

**Methods:**

This cross-sectional study analyzed electronic prescriptions of patients admitted to the intensive care unit of a teaching hospital between January 1 and March 31, 2014 and assessed potential drug-drug interactions associated with antimicrobial drugs. Antimicrobial drug consumption levels were expressed in daily doses per 100 patient-days. The search and classification of the interactions were based on the Micromedex^®^ system.

**Results:**

The daily prescriptions of 82 patients were analyzed, totaling 656 prescriptions. Antimicrobial drugs represented 25% of all prescription drugs, with meropenem, vancomycin and ceftriaxone being the most prescribed medications. According to the approach of daily dose per 100 patient-days, the most commonly used antimicrobial drugs were cefepime, meropenem, sulfamethoxazole + trimethoprim and ciprofloxacin. The mean number of interactions per patient was 2.6. Among the interactions, 51% were classified as contraindicated or significantly severe. Highly significant interactions (clinical value 1 and 2) were observed with a prevalence of 98%.

**Conclusion:**

The current study demonstrated that antimicrobial drugs are frequently prescribed in intensive care units and present a very high number of potential drug-drug interactions, with most of them being considered highly significant.

## Abstract (pt)

**Objetivo:**

Avaliar a existência de interações medicamentosas potenciais na unidade de terapia intensiva de um hospital, com foco nos antimicrobianos.

**Métodos:**

Estudo transversal, que analisou prescrições eletrônicas de pacientes da unidade de terapia intensiva de um hospital de ensino, avaliando potenciais interações medicamentosas relacionadas aos antimicrobianos, entre 1º de janeiro e 31 de março de 2014. O consumo dos antimicrobianos foi expresso em dose diária definida por 100 pacientes-dia. A busca e a classificação das interações foram realizadas com base no sistema Micromedex^®^.

**Resultados:**

Foram analisadas prescrições diárias de 82 pacientes, totalizando 656 prescrições. Do total de medicamentos prescritos, 25% eram antimicrobianos, sendo meropenem, vancomicina e ceftriaxona os mais prescritos. Os antimicrobianos mais consumidos, segundo a metodologia de dose diária definida por 100 pacientes-dia, foram cefepime, meropenem, sulfametoxazol + trimetoprima e ciprofloxacino. A média de interações por paciente foi de 2,6. Entre as interações, 51% foram classificadas como contraindicadas ou de gravidade importante. Destacaram-se as interações altamente significativas (valor clínico 1 e 2), com prevalência de 98%.

**Conclusão:**

Com o presente trabalho verifica-se que os antimicrobianos são uma classe frequentemente prescrita na unidade de terapia intensiva, apresentando elevada quantidade de interações medicamentosas potenciais, sendo a maior parte das interações considerada altamente significativa.

## INTRODUCTION

Adverse drug events (ADE) are a global concern for public policy makers, healthcare professionals and populations because they are very frequent and increase the morbidity and mortality of patients, thus constituting a public health problem.^(1,2)^ Complications associated with the use of drugs are the most common type of adverse event during hospitalization, representing 3-5% of adverse drug reactions that can be prevented in hospitals.^(3)^ Only 25% of ADE are unpredictable or caused by allergic reactions. In most cases (> 70%), the ADE is associated with the dose of the administered drug.^(4)^

ADE includes adverse drug reactions and medication errors (MEs).^(1)^ The World Health Organization defines an adverse drug reaction as any noxious, unintended and unwanted response to a drug that occurs at doses normally used for treatment, prophylaxis or diagnosis.^(5)^ According to Iyer et al., over 30% of all adverse drug reactions occur due to drug interactions, which are considered potentially preventable with early detection, highlighting the importance of early identification.^(6)^

Despite the growing concern among healthcare professionals regarding patient safety, preventable errors still occur frequently, especially in complex environments such as intensive care units (ICU).^(4)^ Due to the severity of their diseases, ICU patients in critical condition often require the administration of drugs with an increased risk of adverse reactions; therefore, they are more vulnerable to ADE. Moreover, the administration of multiple drugs that usually occurs in ICU increases the incidence of ADE.^(4)^

The ICU is the hospital area characterized by the complex treatments given to patients in critical condition who require intensive care. Drug consumption in ICUs is high, and the average number of prescription items per patient can reach 15 drugs.^(7)^ Patients admitted to the ICU are exposed to prolonged treatment protocols, and most of these patients receive some antimicrobial treatment during their hospital stay.^(8-10)^

Currently, antimicrobial drugs are among the most commonly prescribed drugs in hospitals for both treatment and prophylaxis. Approximately 30% of hospital pharmacy costs are associated with the use of these drugs.^(10,11)^ The incidence of infections with resistant microorganisms is becoming increasingly common in hospital environments, especially in ICUs.^(12)^

A drug interaction occurs when the effects and/or toxicity of a certain drug are altered by another drug. Regardless of whether they result in positive (increased efficacy) or negative effects (decreased efficacy, toxicity or idiosyncrasy), the interactions are generally unpredictable and undesirable in pharmacotherapy.^(13)^ "Potential drug-drug interaction" is the term that is used to refer to the possibility a drug has to alter the effects of another drug when they are simultaneously administered. Such interactions may occur either before their administration (physical-chemical interaction or incompatibility) or after their administration.^(7)^

The potential for the incidence and the severity of drug interactions depend on several factors. The risk of drug-drug interactions increases with both the number of drugs used and the physiological changes associated with aging. In addition, the presence of potential drug-drug interactions (PDDI) is directly associated with increased length of stay in the ICU.^(7,13,14)^

Studies addressing the use of drugs are performed because of health concerns and seek to generate information that can be used to positively transform the observed reality. These studies are essential to detect, analyze and solve the problems arising from the improper use of drugs.^(15,16)^

This study aimed to evaluate the incidence of potential drug-drug interactions in the intensive care unit of a teaching hospital and focused on antimicrobial drugs.

## METHODS

This study was conducted in the Pharmacy Department of the *Hospital Universitário da Universidade Federal de Juiz de Fora*, Juiz de Fora (MG - Brazil), a reference center for providing care for the Unified Health System (*Sistema Único de Saúde* - SUS) patients. This hospital had an installed and occupancy capacity of 140 beds. The Pharmacy Department analyzed in this study was not performing the clinical assessment of the admitted patients because this service was undergoing restructuring.

This cross-sectional study assessed the prescription of drugs, focusing on potential drug-drug interactions in a hospital ICU. The study analyzed the daily electronic prescriptions (total analyzed prescriptions = mean length of hospital stay x total number of patients admitted during the study period) using the Management System for Teaching Hospitals of patients admitted to the hospital ICU, whose capacity was 9 beds. This study was approved by the hospital Research Ethics Committee (number 711.426). The Free and Informed Consent form was waived because the study involved data review.

Prescriptions for patients admitted to the ICU from January 1 to March 31, 2014 that met the following inclusion criteria were selected: containing two or more drugs, with at least one of them being an antimicrobial drug, and patients aged more than 18 years and hospitalized in the ICU for at least 24 hours. All daily prescriptions from the ICU that were delivered to the pharmacy meeting these criteria were analyzed. There were no exclusion criteria.

The use of each antimicrobial drug was expressed as the defined daily dose (DDD) per 100 patient-days for 3 months. The classification Anatomical Therapeutic Chemical Classification System with Defined Daily Doses (ATC/DDD) of the World Health Organization,^(17)^ version 2015, was used to calculate the number of DDD for each antimicrobial drug. DDD differs for each drug and represents the "assumed average maintenance dose per day for a drug used for its main indication."^(16)^ DDD is a fixed unit of measurement regardless of price and dosage form and allows trends in drug consumption to be evaluated.^(18)^ It is noteworthy that the DDD is not a recommended dose but rather a unit of measurement that allows results to be compared.^(16)^ DDD/100 patient-days allows the percentage of the consumption of a certain drug to be estimated for a given period of time and suggests the probability of treating a patient with a particular drug.^(16)^

The amount of drug used (in DDD) was calculated according to the following formula: number of DDD = number of units sold or dispensed x number of dosage forms per unit x amount of active ingredient per dosage form/DDD value.

For the DDD/100 patient-days calculation, the following formula was used: DDD/100 patient-days = number of DDD x 100/occupancy index x number of available beds x time in days.

The search and classification regarding the severity and documentation of PDDIs were based on the Micromedex^®^ system.^(19)^ The PDDIs were also classified according to the clinical value, which correlates the severity of the effect and the documentation of the interactions. The clinical value was classified from 1 to 5, as shown in table 1S (electronic supplementary material). Furthermore, the following recommendations were used to indicate the clinical value of the PDDI: (1) avoid combinations; (2) usually avoid combinations; (3) minimize the risk; (4) no action is required; and (5) no interaction. A clinical value of 1 or 2 indicates a significant PDDI. This classification was performed based on a modified classification described by Tatro.^(20)^

Data were collected, recorded in an electronic spreadsheet and submitted to descriptive statistical analysis in Microsoft Office Excel^®^. For the comparison between specialties (i.e., clinics responsible for the patient) and the incidence of PDDI, the proportion analysis was performed using the chi-square test and was adjusted using Fisher's test. For the comparison between length of hospital stay in the ICU and the mean number of PDDI, the length of stay was stratified into two groups according to their mean, and then the Mann-Whitney test was used. The statistical significance was set at 5%.

## RESULTS

The sample consisted of the medications to be administered over a period of 24 hours to 82 patients during their hospitalization in the ICU, totaling 656 prescriptions. Among the patients, 54% were male. The patients' age ranged from 18 to 89 years (mean age 60 ± 18 years), and 50% of patients were 60 years or older. The analysis of the entire sample revealed that a total of 864 drugs were dispensed and that 131 different drugs were observed. The patients received 3-24 drugs, with a mean of 10.5 ± 4.7 drugs. The length of stay in the ICU was 2 to 48 days with a mean of 8 ± 7.85.

Regarding the origin of the patients admitted in the ICU during the study period, 30 (37%) were from the surgery department, 29 (35%) from the clinical services, and 23 (28%) from other hospitals. The pulmonology service was responsible for the majority of the hospitalizations (17%), followed by surgery (including general, thoracic, vascular, plastic and head and neck surgeries) (15%), gastroenterology (12%), nephrology/urology (11%), neurology (7%), and internal medicine (7%).

The most commonly prescribed drugs in the sample were injectable omeprazole (71%), subcutaneous heparin (63%), injectable norepinephrine (50%), injectable bromopride (43%) and injectable meropenem (38%). Intravenous was the most widely used route of administration (71%), followed by oral/nasogastric tube (24%), subcutaneous (4%) and intramuscular (1%) routes. Antimicrobial drugs represented 25% of all prescription drugs, with meropenem (38%), vancomycin (29%), ceftriaxone (20%) and metronidazole (18%) being the most prescribed drugs (Table 2S - electronic supplementary material). Regarding the antimicrobial agents, the most frequently used route of administration was intravenous (92%), followed by oral (7%) and nasogastric tube (1%). Table 3S (electronic supplementary material) shows the use of antimicrobial drugs expressed in DDD/100 patient-days calculated for each of the antimicrobial drugs used in the study period. According to the DDD approach, the most commonly used antimicrobial drugs were cefepime, meropenem, sulfamethoxazole + trimethoprim and ciprofloxacin, which corresponded to 24.01 DDD/100 patient-days, 21.95 DDD/100 patient-days, 12.64 DDD/100 patient-days, and 12.47 DDD/100 patient-days, respectively.

Regarding the drug interactions associated with antimicrobial drugs, 98 incidences were found in 46% (36) of the patients. Among these 98 incidences, 58 different types were observed. The number of interactions per patient ranged from 1 to 13, with a mean of 2.6 PDDI incidences per patient. Patients with lower PDDI incidences (below 2.6) presented lengths of stay in the ICU similar to those with higher PDDI incidences (9.04 ± 7.2 days and 15.08 ± 13.75 days, p = 0.191).

The 10 most frequent drug interactions are shown in table 1. The most frequent PDDIs occurred with fluconazole-omeprazole (9 patients presented the interaction at least once) and ampicillin + sulbactam-omeprazole (7 patients presented the interaction at least once).

**Table 1:** The most frequent potential drug-drug interactions classified by clinical value, severity and documentation

**Potential drug-drug interactions**	Clinical value*	**Severity**	**Documentation**	**N**
Fluconazole - omeprazole	2	Moderate	Excellent	9
Ampicillin + sulbactam - omeprazole	2	Moderate	Fair	7
Moxifloxacin - hydrocortisone	2	Moderate	Excellent	4
Ciprofloxacin - fentanyl	1	Severe	Fair	3
Ciprofloxacin - haloperidol	1	Severe	Fair	3
Fluconazole - amiodarone	1	Contraindicated	Fair	3
Fluconazole - fentanyl	1	Severe	Fair	3
Fluconazole - midazolam	2	Moderate	Excellent	3
Fluconazole - prednisone	2	Moderate	Good	3

N - Number of patients who presented the interaction at least once.

* 1: avoid combinations; 2: usually avoid combinations.

PDDI incidences according to specialty are shown in table 2. There were no significant differences between PDDI incidences by specialty (p = 0.622), i.e., PDDI incidences were similar among the ICU patients analyzed in this study.

**Table 2:** Potential drug-drug interactions and the number of patients who presented the interactions by specialty

Specialty	Patients admitted to the ICU	Patients who presented PDDI	Number of PDDI	p value
Pulmonology	17% (14)	16% (6)	18	0.622
Infectious diseases	5% (4)	8% (3)	17	
Neurology	7% (6)	11% (4)	12	
Surgery*	15% (12)	11% (4)	9	
Nephrology/urology	11% (9)	16% (6)	8	
Gastroenterology	12% (10)	11% (4)	8	
Hepatology	5% (4)	5% (2)	8	
Rheumatology	4% (3)	5% (2)	5	
Internal medicine	7% (6)	8% (3)	4	
Cardiology	2% (2)	3% (1)	4	
Intensive care	4% (3)	5% (2)	3	
Proctology	6% (5)	3% (1)	2	
Others	4% (4)	-	-	
Total	82	38	98	

ICU - intensive care unit; PDDI - potential drug-drug interactions.

* General, plastic, chest, head and neck surgeries.

The frequencies of the PDDI incidences according to the severity classification, documentation and clinical value are shown in table 3. It is noteworthy that approximately 51% of the recorded PDDI were classified as contraindicated or severe and that approximately 98% of them were classified with a clinical value of 1 or 2, which is considered highly significant.

**Table 3:** Potential drug-drug interactions according to their severity, documentation and clinical value

	Frequency (%)
Severity	
Contraindicated	9 (9.18)
Important	41 (41.84)
Moderate	46 (46.94)
Secondary	2 (2.04)
Documentation	
Excellent	26 (26.53)
Good	21 (21.43)
Fair	51 (52.04)
Clinical value*	
1	50 (51.02)
2	46 (46.94)
3	2 (2.04)

* 1: avoid combinations; 2: usually avoid combinations; 3: minimize the risk.

## DISCUSSION

This study revealed that 46% of patients presented PDDI associated with antimicrobial drugs. Among these PDDI, 51% were classified as contraindicated or significantly severe. Highly significant interactions (i.e., with a clinical value of 1 or 2) were observed with a prevalence of 98%. These types of interactions should be avoided as much as possible.

The current study demonstrated a mean of 10.5 prescribed drugs per patient. This result is similar to the one reported by Cedraz et al. in a study performed in a public hospital located in the city of Feira de Santana (BA, Brazil), who found a mean of 11.96 prescribed drugs per patient. The elevated number of prescription drugs for ICU patients represents risk because it is directly proportional to the development of drug interactions and adverse effects, which then increase the length of the hospital stay.^(21)^

A total of 71% of the drugs were intravenously administered; when only antimicrobial drugs were considered, this delivery route represented 92% of the administrations. The intravenous route is notoriously the most commonly used method in the ICU due to the severity of the clinical status of patients, who require a fast administration route to obtain immediate clinical effects.^(10)^ In addition to having a rapid effect, the intravenous route offers immediate access to the circulatory system and allows the administration of high doses and high concentrations through a central route. However, the intravenous route also has disadvantages associated with the risk of tissue infiltration or extravasation, drug interactions by direct contact, risk of contamination and pyrogenicity.^(7)^

Omeprazole had higher potential interactions with the studied antimicrobial drugs and accounted for 16.32% of the PDDI. These interactions are considered highly significant (clinical level 2), which indicates that this drug needs to be rationally used to prevent adverse events. Furthermore, it was the most widely prescribed drug and was presented in 71% of the prescriptions. Its extensive use can be explained by the high number and variety of drugs used in the ICU that promote symptoms involving the gastrointestinal tracts of patients with adverse drug reactions.^(22)^ The use of proton pump inhibitors has demonstrated effectiveness in the treatment of peptic ulcers in ICU patients.^(23)^

The ICU is characterized by the elevated consumption of antimicrobial drugs, which reflects the severe condition of patients and the high infection rates.^(11)^ The results of the current study demonstrated this fact, revealing that antimicrobial drugs represent 25% of all prescriptions. Considering the most commonly prescribed antimicrobial drugs, a study performed by Curcio et al. investigated the prescription of antibiotics in 43 ICUs in Latin America, and their results were similar to the ones found in the present study: carbapenems (imipenem or meropenem) were the most frequently prescribed antibiotics (22%), followed by vancomycin (15%), piperacillin-tazobactam (12.5%) and broad-spectrum cephalosporins (12%).^(24)^

Regarding the consumption of antimicrobial drugs expressed in DDD/100 patient-days for three months, DDDs were calculated for each antimicrobial drug used in the period of data collection, enabling a better view of what was consumed by the hospital. Most studies using the DDD approach performed the calculations for antimicrobial drug groups and used the DDD per bed-days. The terms "bed-days" and "patient-days" are different, as bed-days means the "bed available to the patient for 1 day", while patient-days means "a patient occupying a bed for 1 day." Currently, studies evaluating the use of antimicrobial drugs in ICUs use DDD per patient-days because it estimates the use of a particular drug by one patient.^(16)^

In the current study, the most commonly used antimicrobials were cefepime, meropenem, sulfamethoxazole + trimethoprim and ciprofloxacin. A study performed by Santos et al. also used the DDD per patient-day approach, but the results were expressed according to antimicrobial drug groups. These authors evaluated the consumption of these drugs in three non-specialized ICUs (2 public and in 1 private) in Brasilia, the Federal District of Brazil, and found significant differences in consumption between hospitals.^(25)^

The comparison between different studies reveals that each hospital has its own characteristics and that both the type of antimicrobial drug and the amount consumed can be different and can involve a number of local factors that contribute to prescribe a specific antimicrobial drug.^(18)^ Despite the fact that the ATC/DDD approach has been designed to allow comparisons between the consumed drugs, one must take into account that each location has its own characteristics that must be considered to validate the comparisons,^(18)^ such as in comparisons using data from the same hospital in different periods, which may indicate a drug consumption trend.

The incidence rates of drug interactions in ICUs are much higher than the general rates observed in the entire hospital, mainly due to the large number of drugs administered and the profile of the patients admitted in this sector.^(7)^ Approximately 51% of the recorded PDDIs were classified as contraindicated or severe, which may be associated with life-threatening conditions and/or require medical intervention to reduce or avoid serious adverse reactions.

Among them, special attention should be paid to PDDIs involving simvastatin (incidence of 10% among the PDDIs with the clinical value of 1), which should not be used in combination with azithromycin, ciprofloxacin, clarithromycin and fluconazole due to the increased risk of myopathy or rhabdomyolysis. Regarding the forms of muscular involvement, myopathy is defined as any muscle discomfort; rhabdomyolysis is characterized by muscle weakness, significant elevation of creatine phosphokinase (CPK), usually more than ten times the upper limit of normal, increased serum creatinine, myoglobinuria and acute renal failure.^(26)^ Despite rhabdomyolysis being unusual, it is the most serious adverse effect observed in the lipid-lowering therapy with statins and is potentially fatal.^(26)^

Concomitant uses of fluconazole-amiodarone, fluconazole-ondansetron, fluconazole-ritonavir and metronidazole-amiodarone are combinations that represent 37.5% of the interactions with clinical values of 1 and should also be avoided because of the increased risk of cardiotoxicity (QT prolongation). The QT interval is the time between the onset of ventricular depolarization and the end of the T wave (ventricular repolarization) and therefore represents the total duration of the ventricular electrical activity. Long QT syndrome is quite common in ICU patients, is usually triggered by drugs and/or electrolyte disturbances and can negatively affect the patient's outcome.^(27)^ ICU patients with QT prolongation have longer hospital stays and higher hospital mortality rates compared to ICU patients without the condition.^(27)^ Identification and proper monitoring of high-risk patients are essential to prevent the PDDIs that can cause QT interval prolongation and, therefore, hospital deaths.^(27)^

When the use of antimicrobial drugs that can potentially interact with other drugs is necessary, analyses of possible drug interaction effects and the careful monitoring of the patient undergoing therapy are recommended. It is known that measures other than suspending the combination can control most PDDIs, such as dosage adjustment and the monitoring of the possible adverse events, with benefits and risks being individually assessed.^(3)^

The quality of the prescriptions is essential to preserve the effectiveness of the available antimicrobial drugs, which highlights the important role of healthcare professionals in improving the current conditions.^(18)^ The work of a multidisciplinary clinical team with the participation of the clinical pharmacists is important, as the pharmaceutical interventions may contribute to reduce preventable adverse events.

The current study has a number of limitations. Some of the existing confounding factors were not controlled in this study. The drug-drug combinations analyzed among the drugs to be administered over a 24-hour period suggest that the drugs were simultaneously used, but the administrations may have occurred at different times within the day. Although the study classified the interactions according to their severities and levels of evidence, the actual incidence of the interaction has not been investigated. In addition, the number of described PDDIs was record from the first prescription during the hospitalization period; the number of days that these PDDIs may have occurred was not recorded. Finally, this study was single-centered in nature, with the inclusion of small numbers of patients. Furthermore, this population was not properly characterized.

The high number of PDDIs among critically ill patients indicates that further studies are necessary. In addition, the results show that the healthcare professionals involved in the care of patients admitted to the ICU need to improve their knowledge about the risks and benefits involving drug interactions, clinical management and the actual occurrence of these interactions.

## CONCLUSION

The current study demonstrated that antimicrobial drugs are frequently prescribed in the intensive care unit and present very high numbers of potential drug-drug interactions, with most of them being considered highly significant.
