# Supplementary material for: Adverse events caused by potential drug-drug interactions in an intensive care unit of a teaching hospital
Source: Rev Bras Ter Intensiva. 2015 Oct-Dec;27(4):353–9. doi: 10.5935/0103-507X.20150060 (PMC4738821; doi:10.5935/0103-507X.20150060)
Supplement: Supplementary file 1 [file rbti-27-04-0353-suppl01.pdf]

Adverse events caused by potential drug-drug interactions in an intensive care unit of a teaching hospital

Eventos adversos por interações medicamentosas potenciais em unidade de terapia intensiva de um hospital de ensino

Mariana Macedo Alvim[1], Lidiane Ayres da Silva[1], Isabel Cristina Gonçalves Leite[1], Marcelo Silva Silvério[1]

Table 1S - Clinical values of the potential drug-drug interactions

Severity	Documentation			
	Excellent	Good	Fair	Unknown
Contraindicated	1	1	1	5
Important	1	1	1	5
Moderate	2	2	2	5
Secondary	3	3	4	5
Unknown	5	5	5	5

Modified from: Tatro DS. Drug interaction facts. 2nd ed. St. Louis: Facts & Comparisons; 2013. Recommendations: 1 - avoid combinations; 2 - usually avoid combinations; 3 - minimize the risk; 4 - no action is required; 5 - no interaction.

Table 2S - Number of patients who have been prescribed antimicrobial drugs

Most frequently prescribed antimicrobial drugs	Prescription N (%)
Meropenem	31 (38)
Vancomycin	24 (29)
Ceftriaxone	16 (20)
Metronidazole	15 (18)
Ciprofloxacin	14 (17)
Cefepime	13 (16)
Moxifloxacin	11 (13)
Fluconazole	9 (11)
Ampicillin + sulbactam	8 (10)
Amphotericin B	6 (7)
Piperacillin + tazobactam	6 (7)
Sulfamethoxazole + trimethoprim	6 (7)
Teicoplanin	6 (7)
Metronidazole	5 (6)
Amikacin	4 (5)
Azithromycin	4 (5)
Cefazolin	4 (5)
Clindamycin	4 (5)
Polymyxin B	4 (5)
Amoxicillin+ clavulanate	3 (4)
Ceftazidime	3 (4)
Others	18 (22)

Table 3S - Use of antimicrobial drugs expressed in defined daily doses per 100 patient-days

Antimicrobial drugs	DDD per 100 patient-days
Cefepime	24.01
Meropenem	21.95
Sulfamethoxazole + trimethoprim	12.64
Ciprofloxacin	12.47
Amphotericin B	12.31
Vancomycin	12.19
Ampicillin + sulbactam	10.26
Metronidazole	9.86
Ceftriaxone	9.76
Fluconazole	7.73
Ampicillin	7.35
Moxifloxacin	6.46
Teicoplanin	3.55
Piperacillin + tazobactam	3.29
Polymyxin B	3.12
Clindamycin	2.53
Amikacin	2.17
Azithromycin	2.11
Tenofovir	1.40
Oxacillin	1.27
Ceftazidime	1.20
Amoxicillin + clavulanate	1.14
Fosamprenavir	1.08
Lamivudine	1.08
Ganciclovir	1.01
Cefazolin	0.89
Norfloxacin	0.51
Clarithromycin	0.06
Ritonavir	0.01

DDD - defined daily doses.
